# Patterns of physical co-/multi-morbidity among patients with serious mental illness: a London borough-based cross-sectional study

**DOI:** 10.1186/1471-2296-15-117

**Published:** 2014-06-11

**Authors:** Charlotte Woodhead, Mark Ashworth, Peter Schofield, Max Henderson

**Affiliations:** 1 Department of Psychological Medicine, Institute of Psychiatry, King’s College London, London, UK; 2 Department of Primary Care & Public Health Sciences, School of Medicine, King’s College London, London, UK; 3 Division of Health & Social Care Research, School of Medicine, King’s College London, London, UK

**Keywords:** Serious mental illness, Mental health, Physical health, Comorbidity, Multimorbidity

## Abstract

**Background:**

Serious mental illness (SMI) is associated with elevated mortality compared to the general population; the majority of this excess is attributable to co-occurring common physical health conditions. There may be variation within the SMI group in the distribution of physical co/multi-morbidity. This study aims to a) compare the pattern of physical co- and multi-morbidity between patients with and without SMI within a South London primary care population; and, b) to explore socio-demographic and health risk factors associated with excess physical morbidity among the SMI group.

**Methods:**

Data were obtained from Lambeth DataNet, a database of electronic patient records derived from general practices in the London borough of Lambeth. The pattern of 12 co-morbid common physical conditions was compared by SMI status. Multivariate ordinal and logistic regression analyses were conducted to assess the strength of association between each condition and SMI status; adjustments were made for potentially confounding socio-demographic characteristics and for potentially mediating health risk factors.

**Results:**

While SMI patients were more frequently recorded with all 12 physical conditions than non-SMI patients, the pattern of co-/multi-morbidity was similar between the two groups. Adjustment for socio-demographic characteristics – in particular age and, to a lesser extent ethnicity, considerably reduced effect sizes and accounted for some of the associations, though several conditions remained strongly associated with SMI status. Evidence for mediation by health risk factors, in particular BMI, was supported.

**Conclusions:**

SMI patients are at an elevated risk of a range of physical health conditions than non-SMI patients but they do not appear to experience a different pattern of co-/multimorbidity among those conditions considered. Socio-demographic differences between the two groups account for some of the excess in morbidity and known health risk factors are likely to mediate the association. Further work to examine a wider range of conditions and health risk factors would help determine the extent of excess mortality attributable to these factors.

## Background

The UK policy paper, ‘No Health Without Mental Health’ published in 2011 [1] emphasised severe mental illness across all of its six outcome objectives. The third objective acknowledged that the physical health of people with severe mental illness should be improved to address inequalities in mortality and morbidity.

Patients with severe mental illness (SMI) such as schizophrenia and bipolar affective disorder die younger than the background population [2-4]. This mortality gap has been estimated at 10–18 years for women and 8–19 years for men [2,4-6]. These excess deaths are largely attributed to common physical disorders, most notably coronary heart disease and stroke rather than ‘unnatural’ deaths from violence or suicide [2,3,6-8]. The precise reasons for this excess mortality have yet to be elucidated. There are a range of inter-related risk factors including treatment-related factors [9,10], health-related behaviours of patients with SMI [11,12], access to and utilisation of healthcare by this patient group, and the delivery of inferior healthcare to those that do [13-15], and disease-related factors [16].

Behind the headline areas regarding mortality, patients with SMI experience a range of chronic physical health problems which may interfere with quality of life. Their outcomes when they receive treatment are often poor. Studies within secondary care are limited by their inability to compare results with the non-SMI population. Yet there are few large-scale primary care studies of the physical health of patients with SMI with the exception of Smith et al. [17].

Our study aims to extend previous work by looking in detail at the physical health of an entire London borough. We aimed to investigate whether patterns of physical health comorbidity differed between patients with and without SMI, and further, to examine the extent to which this may be accounted for by individual factors, in particular ethnicity.

## Methods

The London borough of Lambeth is home to nearly 303100 residents and is the 29^th^ most deprived borough in the UK [18]. Lambeth is extremely diverse; data from the 2011 Census reveal it to have the highest proportion in the country of: Portuguese and South American born people; mixed race White and Black African people; people from multiple mixed ethnic backgrounds; people from non-Caribbean and non-African black backgrounds; and, the second highest proportion of Black Caribbean people in the country.

Data were extracted from the computerised medical records of all except one GP practices (*n* = 48) within Lambeth, as part of Lambeth DataNet (LDN) (the missing GP practice has an incompatible IT system). LDN is a primary care database which collects anonymised data on GP consultations, prescriptions, and Quality and Outcomes Framework (QOF) data on clinical targets - as well as certain demographic information: age, gender, self-identified ethnicity, and truncated postcode. LDN was started in 2004 as a local resource to improve ethnicity recording and to explore local health inequalities, particularly those based on ethnicity or social deprivation. LDN currently covers a population of 366,317 registered patients. This exceeds the total Lambeth population recorded in the Census, likely due to the cross-boundary registration of patients not living in Lambeth, list inflation, and the temporary residence of students in the Lambeth area from surrounding Universities.

Those with a serious mental illness (SMI) are identified in LDN as part of the QOF initiative to include those with bipolar affective disorder, schizophrenia and schizoaffective disorder, though data are not available to distinguish between these diagnoses.

### Measures

#### Socio-demographic characteristics

Information was collected on: age, gender, ethnicity and index of multiple deprivation score [18]. IMD scores were divided into quintiles, such that comparisons were made with the registered population of Lambeth rather than nationally. Previous research within South East London has found differences in mental health outcomes between ethnic groups that are often otherwise aggregated in analyses, notably between Black Caribbean and Black African groups [19]. Therefore the following sub-categories of ethnicity were examined: British/mixed British; Black African; Back Caribbean; Asian/Asian British; mixed; other White; other Black; and, other.

#### Physical health conditions

QOF requires GPs to keep a register of patients with a number of long term health conditions indicated with binary (yes/no) variables. Of the 19 conditions covered by QOF, 14 were available for the current study and 12 were included in analyses. Dementia and depression were excluded due to the focus on physical health conditions and potential overlap with SMI status. Conditions retained for analysis were: hypertension (BP), epilepsy, diabetes mellitus (DM), coronary heart disease (CHD), chronic kidney disease (CKD), chronic obstructive pulmonary disorder (COPD), cancer (non-specified), atrial fibrillation, heart failure, stroke, hypothyroidism, and asthma. Clinical areas for which there was no available information were: rheumatoid arthritis (introduced in the 2013/14 guidelines), osteoporosis, learning disability, and palliative care, peripheral arterial disease. These outcomes were not included in the database extraction used here.

#### Health risk factors

Data on patients’ Body Mass Index (BMI) and smoking status were assessed. BMI scores were categorised into ‘underweight’ (BMI <18.5), ‘desirable’ (BMI 18.5-24.99), ‘overweight’ (BMI > =25), ‘obese’ (BMI > =30), and ‘morbidly obese’ (BMI > =40). Smoking status was categorised as ‘smoker’, ‘non-smoker’ and ‘ex-smoker’.

### Statistical analyses

Analyses were carried out using STATA 11 (Stata Corp, 2009). Patients aged below 16 years were excluded. Socio-demographic characteristics and individual health conditions were compared by SMI status using multivariate logistic regression or with ordered logistic regression analyses (BMI and smoking). All models accounted for clustering by GP practice using the cluster option.

Models were adjusted for socio-demographic characteristics and health risk factors as appropriate; adjusted odds ratios (OR) and 95% confidence intervals (CI) are presented. Model fit was assessed using Hosmer and Lemeshow’s goodness of fit test with the lfit command.

To investigate the pattern of health conditions experienced by SMI status, individual health conditions were listed and ranked according to the proportion of SMI patients registered with each outcome. Values were then graphically compared by SMI status, illustrating health conditions (in the order of frequency determined by the SMI group), against the proportion of the GP patient population registered with each outcome.

Data summarising the combination of health conditions registered for each patient were collapsed separately by SMI status; frequencies of discrete combinations of conditions were generated and ordered from the most to least frequent. The ten most frequent combinations of health conditions are presented by SMI status.

### Ethical approval

National Research Ethics Committee: concerning the use of general practice data and practice specific quality indicators. Chairman’s action.

## Results

Among patients aged 16 years or more (*n* = 308,643), 1.41% were recorded with a serious mental illness (SMI).

### Socio-demographic characteristics

SMI patients were more likely to be male and older than non-SMI patients (mean age 48.7 years vs 40.6 years, *p* < .001 (data not shown)). There was considerable missing ethnicity data (~20%) yet substantially lower proportion of SMI patients had their ethnicity reported as ‘unknown’ or ‘not stated’ than non-SMI patients (11.1% vs. 19.0%, data not shown). Significant differences in SMI status were found for ethnicity (*p* < .001). Specifically, a greater proportion of ‘Black African’, ‘Black Caribbean’, ‘mixed’, and ‘other Black’ groups were identified with SMI than ‘British/mixed British’ patients; this was particularly pronounced among Black Caribbean’s, with over twice the odds of being recorded with an SMI. ‘Other White’ and ‘other’ groups had lower odds of being recorded with an SMI. Greater deprivation was positively associated with SMI status (*p* < .001); nearly 35.9% of SMI patients were in the most deprived quintile compared to 30.7% of non-SMI patients (Table 1).

**Table 1:** Socio-demographic characteristics of patients by SMI status

	**Non-SMI (16+ yrs) *n*** **= 304,297**		**SMI (16+ yrs) *n*** **= 4,346**		**Adjusted OR**^**1**^
	***n***	**%**	***n***	**%**	**(95% CI)**
**Age group (years)**					
16-24	34718	11.4	146	3.4	1.00
25-34	94479	31.1	717	16.5	1.98 (1.63 - 2.41)^***^
35-44	72060	23.7	976	22.5	3.57 (2.94 - 4.32)^***^
45-54	49334	16.2	1118	25.7	5.63 (4.65 – 6.81)^***^
55-64	26419	8.7	679	15.6	6.65 (5.45 - 8.10)^***^
65+	27287	9.0	710	16.3	5.95 (4.88 - 7.24)^***^
**Sex**					
Female	148778	48.9	1914	44.0	1.00
Male	155500	51.1	2432	56.0	1.31 (1.23 - 1.40)^***^
**Ethnicity (w/data)**					
British/mixed British	86815	35.2	1292	33.5	1.00
Black African	27429	11.1	514	13.3	1.19 (1.07 - 1.32)^**^
Black Caribbean	21206	8.6	789	20.4	2.11 (1.93 - 2.32)^***^
Asian/Asian British	18968	7.7	261	6.8	1.00 (0.87 - 1.14)
Mixed	11050	4.5	232	6.0	1.61 (1.40 - 1.86)^***^
Other White	65017	26.4	549	14.2	0.61 (0.55 - 0.68)^***^
Other Black	7275	3.0	167	4.3	1.60 (1.36 - 1.89)^***^
Other	8806	3.6	59	1.5	0.47 (0.36 - 0.61)^***^
**IMD quintile (in Lambeth)**					
1st (least deprived)	61365	20.2	609	14.0	1.00
2^nd^	61316	20.2	806	18.6	1.39 (1.24 - 1.56)^***^
3^rd^	28723	9.4	449	10.3	1.60 (1.40 - 1.83)^***^
4^th^	59354	19.5	920	21.2	1.55 (1.39 - 1.74)^***^
5th (most deprived)	93539	30.7	1562	35.9	1.62 (1.46 - 1.79)^***^

Numbers (*n*), percentages (%), and adjusted odds ratios (OR) with 95% confidence intervals (CI) are shown.

^1^Adjusted for all other variables.

**p < .01 ***p < .001.

### Health risk factors and behaviours

A lower proportion of SMI patients had missing data for BMI and smoking status compared to non-SMI patients (6.8% vs 23.0% and 0.9% vs 6.8% respectively, data not shown). All BMI categories were associated with SMI status, compared to the reference group (*p* < .001). Adjustment for ethnicity and age and to a slight extent, deprivation, reduced the strength of association between SMI status and BMI; though the morbidly obese group remained 2.5-fold more likely to be recorded with SMI than the reference group (Table 2).

**Table 2:** BMI group and smoking status by SMI status

	**Non-SMI (16+ yrs) *n*** **= 304,297**		**SMI (16+ yrs) *n*** **= 4,346**		**Unadjusted OR (95% CI)**	**Adjusted OR**^**1 **^**(95% CI)**
	***N***	**%**	***n***	**%**		
**BMI group**						
Underweight	8118	3.5	113	2.8	1.22 (1.01 - 1.47)^*^	1.29 (1.05 - 1.58)^*^
Desirable	116991	50.2	1337	33.3	1.00	1.00
Overweight	68312	29.3	1292	32.2	1.65 (1.50 - 1.83)^***^	1.31 (1.18 - 1.45)^***^
Obese	35465	15.2	1105	27.5	2.73 (2.47 – 3.01)^***^	1.95 (1.78 - 2.14)^***^
Morbidly obese	4260	1.8	171	4.3	3.51 (2.98 - 4.14)^***^	2.63 (2.23 - 3.09)^***^
**Smoking**						
Smoker	68188	24.1	1883	43.7	2.79 (2.51 – 3.10)^***^	3.19 (2.86 - 3.55)^***^
Non-smoker	170365	60.1	1685	39.1	1.00	1.00
Ex-smoker	44942	15.9	737	17.1	1.66 (1.50 - 1.84)^***^	1.55 (1.38 - 1.75)^***^

Numbers (*n*), percentages (%), and odds ratios (OR) with 95% confidence intervals (CI) are shown.

Ordinal logistic regression. CIs based on robust standard errors, clustered by GP practice.

*p < .05 ***p < .001.

^1^Adjusted for socio-demographic characteristics.

SMI status was significantly associated with being a smoker or an ex-smoker (compared to non-smokers). Adjustment for age and in particular, ethnicity, increased the effect size for smokers but had only a slight impact on that for ex-smokers compared to non-smokers, in the opposite direction (with a reduction in effect size).

### Patterns of co/multi-morbid health problems

**Figure 1:**
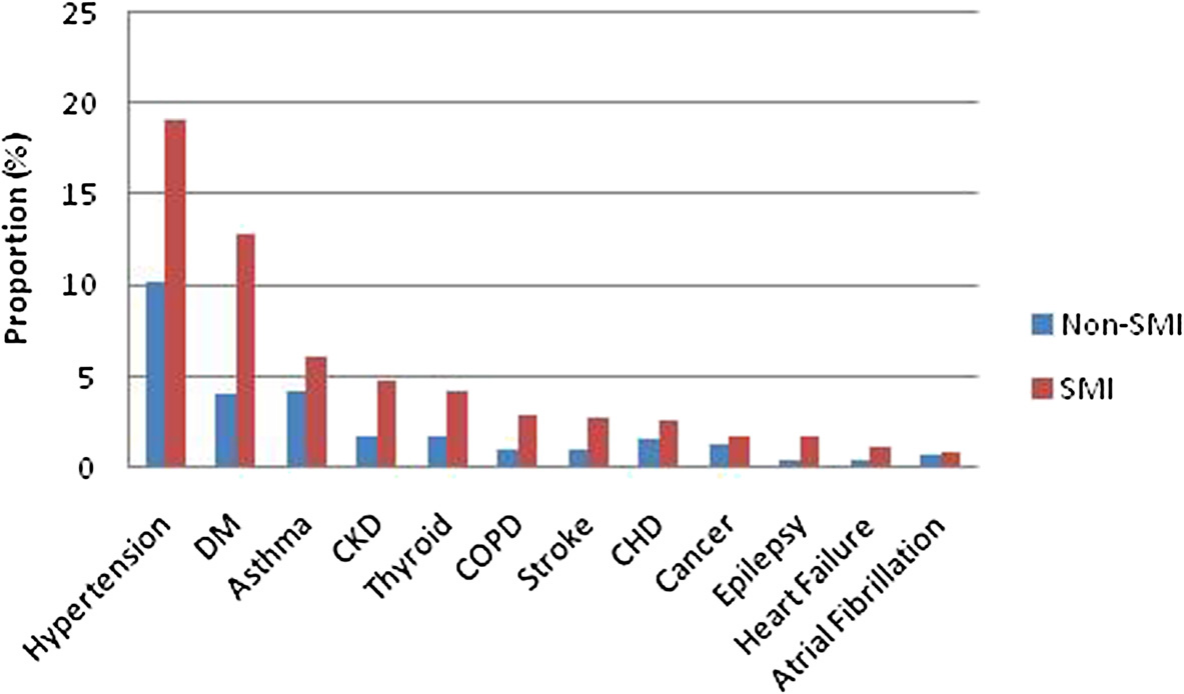
Percentage (%) of SMI and non-SMI patients recorded with each physical health condition.

Figure 1 illustrates the proportion of Lambeth patients recorded with each physical condition by SMI status; more common conditions on the left and rarer conditions to the right. The graph suggests that SMI patients are more commonly recorded with each condition but that the pattern of prevalence is similar among the two groups; the conditions do not differentially cluster in SMI patients compared to non-SMI patients.

Similarly, the frequency with which discrete combinations of physical health problems occurred were similar for the first five most common comorbidity combinations among SMI and non-SMI patients, though differences appeared in the subsequent five combinations (Table 3).

**Table 3:** Ten most common patterns of co/multimorbid physical health problems by SMI status

	**Non-SMI**	**SMI**
1	Hypertension	Hypertension
2	Asthma	Diabetes Mellitus
3	Hypertension + Diabetes Mellitus	Hypertension + Diabetes Mellitus
4	Diabetes Mellitus	Asthma
5	Hypothyroidism	Hypothyroidism
6	Cancer	Chronic kidney disease
7	Hypertension + Chronic Kidney Disease	Hypertension + Chronic kidney disease + Diabetes Mellitus
8	Hypertension + asthma	Chronic obstructive pulmonary disease
9	Hypertension + Coronary heart disease	Hypertension + Chronic kidney disease
10	Chronic obstructive pulmonary disease	Epilepsy

### Association between SMI status and physical health

SMI status was associated with over 2–3 fold greater odds of being recorded with 1, 2 and 3+ health problems. Adjustment for age and to a lesser extent, ethnicity, considerably reduced effect sizes. Age and ethnicity demonstrated multiplicative effects such that the association between SMI status and 3+ physical conditions was rendered non-significant (*p* = 0.476) (Table 4).

**Table 4:** Number of physical health conditions recorded for patients by SMI status

	**Non-SMI (16+ yrs) *n*** **= 304,297**		**SMI (16+ yrs) *n*** **= 4,346**		**Unadjusted OR**	**Adjusted OR**^**1**^	**Adjusted OR**^**2**^
	***N***	**%**	***n***	**%**	**(95% CI)**	**(95% CI)**	**(95% CI)**
**No. health conditions**							
0	249177	81.9	2744	63.8	1.00	1.00	1.00
1	35161	11.6	897	20.6	2.29 (2.07 - 2.54)^***^	1.34 (1.21 - 1.49)^***^	1.13 (1.02 - 1.25)^*^
2	12173	4.0	405	9.3	2.99 (2.59 - 3.45)^***^	1.27 (1.11 - 1.47)^**^	1.06 (0.92 - 1.22)
3+	7786	2.6	270	6.2	3.11 (2.60 - 3.74)^***^	1.06 (0.90 - 1.25)	0.93 (0.78 - 1.10)

Numbers (*n*), percentages (%), and adjusted odds ratios (OR) with 95% confidence intervals (CI) are shown. CIs based on robust standard errors, clustered by GP practice.

*p < .05 **p < .01 ***p < .001.

^1^Adjusted for age, sex, ethnic group, and IMD quintile.

^2^Additionally adjusted for smoking and BMI status.

To assess the extent to which health risk behaviours may be a mechanism for excess comorbidity, further analyses adjusted for smoking and BMI status. Adjustment for BMI status (but not smoking) further reduced effect sizes such that SMI status remained associated only with having one recorded physical health condition, compared to none (*p* = 0.018).

### Individual conditions

SMI status was associated with greater odds of being recorded with every health outcome though adjustment for socio-demographic characteristics markedly reduced the effect sizes - rendering the associations between SMI status and hypertension, asthma, coronary heart disease (CHD), cancer, and atrial fibrillation non-significant. The associations with hypertension and asthma were fully accounted for by the inclusion of both age and ethnicity, which demonstrated multiplicative effects. The associations with CHD, cancer and atrial fibrillation were fully accounted for by age (Table 5).

**Table 5:** Logistic regression analyses assessing the association between physical health conditions and SMI status

	**Non-SMI (*n*** **= 304,297)**		**SMI (*n*** **= 4,346)**		**Unadjusted OR (95% CI)**	**Adjusted OR**^**1 **^**(95% CI)**	**Adjusted OR**^**2 **^**(95% CI)**
	***n***	**%**	***n***	**%**				
BP	30977	10.2	829	19.1	2.08 (1.93 - 2.25)^***^	0.99 (0.88 - 1.12)	0.85 (0.77 - 0.95)
DM	12321	4.1	560	12.9	3.51 (3.20 - 3.84)^***^	2.04 (1.79 - 2.32)^***^	1.77 (1.56 – 2.01)^***^
Asthma	13049	4.3	270	6.2	1.48 (1.31 - 1.67)^***^	1.11 (0.96 - 1.29)	0.96 (0.82 - 1.11)
CKD	5487	1.8	212	4.9	2.79 (2.43 - 3.21)^***^	1.57 (1.31 - 1.88)^***^	1.55 (1.29 - 1.86)^***^
Thyroid	5393	1.8	182	4.2	2.42 (2.08 - 2.82)^***^	1.73 (1.43 - 2.09)^***^	1.60 (1.32 - 1.95)^***^
COPD	3243	1.1	126	2.9	2.77 (2.31 - 3.32)^***^	1.71 (1.35 - 2.15)^***^	1.27 (1.01 - 1.60)^*^
Stroke	3146	1.0	118	2.7	2.67 (2.22 - 3.22)^***^	1.48 (1.17 - 1.87)^**^	1.44 (1.13 - 1.85)^**^
CHD	4927	1.6	112	2.6	1.61 (1.33 - 1.94)^***^	0.94 (0.76 - 1.16)	0.85 (0.70 - 1.05)
Cancer	3867	1.3	76	1.8	1.38 (1.10 - 1.74)^**^	0.82 (0.65 - 1.03)	0.82 (0.65 - 1.04)
Epilepsy	1359	0.5	74	1.7	3.86 (3.05 - 4.89)^***^	2.90 (2.30 - 3.66)^***^	2.72 (2.12 - 3.51)^***^
HF	1427	0.5	53	1.2	2.62 (1.99 - 3.45)^***^	1.54 (1.18 - 2.02)^**^	1.47 (1.09 - 1.97)^*^
AF	2065	0.7	41	0.9	1.39 (1.02 - 1.90)^*^	0.80 (0.56 - 1.15)	0.74 (0.49 - 1.13)

Unadjusted and adjusted odds ratios (OR) and 95% confidence intervals (CI) are shown. CIs based on robust standard errors, clustered by GP practice.

*p < .05 **p < .01 ***p < .001.

^1^Adjusted for age, sex, ethnic group and IMD quintile; ^2^Additionally adjusted for BMI and smoking status.

BP = hypertension; DM = Diabetes mellitus; CKD = Chronic kidney disease; COPD = Chronic obstructive pulmonary disease; CHD = Coronary heart disease; HF = Heart failure; AF = Arial fibrillation.

To determine the extent to which health risk factors may account for excess physical morbidity among SMI patients, additional adjustments for smoking and BMI status were made. This had a slight to moderate influence on effect sizes for COPD (driven by smoking), DM (driven by BMI), hypothyroidism (driven by BMI), epilepsy (driven by BMI and smoking) and heart failure (driven by BMI and smoking) though all remained significantly associated with SMI status. Adjustment for BMI status (but not smoking) reduced the effect size of blood pressure – such that SMI status became protective against high blood pressure.

Model fit was assessed with Hosmer-Lemeshow tests. Prior to addition of health risk behaviours, model fit was adequate only for CHD, CKD, heart failure, atrial fibrillation and epilepsy (*p* > 0.05). For all conditions except epilepsy, addition of health risk behaviours improved the model fit such that model fit was adequate for all models (*p* > 0.05) except epilepsy (*p* < .001). The best fitting model for epilepsy was that which included deprivation score only (*p* = 0.770).

Of those conditions remaining associated with SMI status, diabetes mellitus was the most commonly experienced co-morbid illness among both SMI patients (Table 3).

## Discussion

### Key findings

This study found that the pattern of physical co/multimorbidity among SMI patients did not differ from that among non-SMI patients; nonetheless SMI patients were at increased risk of being recorded with a range of physical health conditions, of being under or overweight, and smoking. After adjustment for socio-demographics and health risk behaviours, SMI status was associated with having one comorbid physical condition compared to none but not with multimorbid (2 or 3+) conditions. Among individual conditions, adjustment for socio-demographic factors (particularly age and ethnicity) and BMI rendered associations between several physical conditions and SMI status non-significant, and reversed the association with hypertension. Significant elevated risks of COPD, stroke, heart failure, chronic kidney disease, hypothyroidism, diabetes and epilepsy among SMI patients remained. Of these conditions, epilepsy demonstrated the strongest association with SMI status, while diabetes was the most commonly experienced comorbid condition among both SMI and non-SMI patients.

### Comparison with previous research

The socio-demographic differences found here between SMI and non-SMI patients are in line with previous studies using comparable data [17,20]; namely, SMI status was associated with older age, male gender, and increased deprivation. Non-white ethnicity is also an established risk factor for SMI [21]. The prevalence of SMI found in the current study (1.4%), is slightly higher than that reported previously for the Lambeth primary care population (1.0%, Pinto et al. [21]).

In a comparable study, Smith et al. [17] compared the physical health of Scottish primary health care patients with schizophrenia (SZ) or related non-organic psychoses to those without and found a stronger association between multimorbidity and SMI status even after adjusting for deprivation. They also reported SMI patients to have 1, 2 and 3+ comorbid health conditions, likely due to the more extensive range of health conditions considered (32 conditions compared to 12 here).

The prevalence of each individual condition was slightly lower among the Scottish study sample compared to that reported here, with the exception of the two most common conditions – hypertension and diabetes – which, alongside CKD, were more prevalent among the SMI group in this study (19% vs 16% and 13% vs 9% respectively). This is likely due to ethnic differences between the samples; for example diabetes and hypertension are more prevalent among African Caribbean, Asian and Black African groups, which constitute a greater proportion of the population of Lambeth compared to Scotland. Other differences may arise as the current study included outcomes that were based on QoF recording. Since QoF outcomes are incentivised, any bias may be expected to increase the recorded prevalence of outcomes in our study. As we found that all except two outcomes were less prevalent in our sample than in the study by Smith et al. based on Read codes, such bias would have led to an underestimation of the difference in the two samples and thus a more conservative interpretation of the difference between the two samples.

Positive associations between SMI status and COPD, thyroid disorders, diabetes and epilepsy were recorded in both studies in regression analyses after adjustment for socio-demographic status - though the associations were stronger in the Lambeth sample despite additional adjustment for ethnicity. Contrary to the current findings, they report no difference in relation to heart failure, stroke or CKD between the two groups; and, unlike the current study, found cancer, CHD, and atrial fibrillation to be significantly less likely among cases. Lastly, hypertension to be less likely after adjustment for socio-demographic characteristics, a finding we replicated only after additional adjustment for BMI.

These differences may be partly due to the differential definition of cases; while they included only SZ and non-organic psychoses, we used a composite measure which also included bipolar affective disorder. Further, we adjusted for ethnicity in addition to age, gender and deprivation. This may have been more important in the current study, given the high proportion of non-white ethnic groups in Lambeth, compared to Scotland, in which according to 2011 Census data, over 95% of the population overall is white. In this study, there was considerable ethnic variation in both SMI status and prevalence of health conditions considered. Ethnicity accounted for much of the association of most health conditions with SMI status, though to a lesser extent than age. Such differences are important to consider in future studies among this population.

Previous research finds those with SMI at greater risk of mortality than unaffected individuals, not accounted for by excess suicide [3,8]. Recent large scale population based studies find much of this excess mortality to be associated with preventable physical health problems, including cardiovascular diseases and cancer [2,4,8], as well as diabetes mellitus [2,22], infectious and respiratory diseases [2,8].

The lack of difference found for CHD and atrial fibrillation in patients with and without SMI, and lower levels of hypertension among SMI patients in the current study, run counter to those findings. It may be interpreted that these outcomes are being underreported in primary care [17]. Alternatively, this finding may be due to excess mortality associated with CHD among the SMI group. This explanation cannot be tested in the current study due to the cross-sectional nature of the data, thus can only provide data on prevalence (which is influenced by both the duration of conditions and the incidence of new cases). Cohort studies providing incidence data do support this hypothesis; for example, Crump et al. [2] report much higher mortality from ischaemic heart disease (and cancer) but no increased risk of diagnoses with these conditions among SMI patients; and, Osborn et al. [20] report an excess of deaths from CHD and stroke among SMI patients compared to control patients.

This study found that SMI patients were less likely to have missing data for ethnicity, BMI and smoking status – possibly suggesting that they do not have reduced access to GP services. Previous research has found an elevated consultation frequency among SMI patients which may account for this finding [15]. Previous work suggests that care offered to SMI patients may be inferior to those without SMI [13]. This finding may also indicate lower consultation rates among non-SMI patients. Future research may elucidate whether the more comprehensive reporting of BMI, ethnicity and smoking is due to greater frequency of access to primary care among SMI patients in this sample; whether or not this translates into better recording of health outcomes (and might account for some of the elevated risk observed here); and, whether or not care offered for physical problems reflects any greater access.

### Health risk behaviours

The impact of adjustment for lifestyle related variables – smoking and BMI - on most conditions found to be associated with SMI status, supports previous research indicating that excess morbidity and mortality is associated with suboptimal lifestyles [6,23]. The findings suggest that interventions to reduce risk behaviours in this group may reduce the risk associated with SMI status for physical health problems. Lifestyle interventions have been found to be effective in the reduction of health risk behaviours [24,25], and to reduce mortality [26]. The remaining excess risk may be partly accounted for by side effects of the use of antipsychotic medications and for genetic commonalities – e.g. as is suggested for CVD risk factors [27].

### Strengths and limitations

The strengths of this study lie in the large sample size and the near complete coverage of GP data in the London borough of Lambeth. The study supports and extends previous findings by examining the pattern of comorbid health conditions by SMI status. The main weaknesses pertain to the cross-sectional nature of the data, the ability to generalise findings beyond Lambeth and the lack of distinction between SMI conditions despite known differences between BPD and SZ and other non-organic psychoses [23]. Further, the contribution of other health risk behaviours to excess physical morbidity, such as diet, exercise and alcohol consumption was not able to be elucidated.

## Conclusions

In all, these data suggest that those with SMI are more likely to be recorded with each condition rather than to experience a different pattern of health conditions than non-SMI patients, and that among the 12 health outcomes included in the current study, the pattern of co/multimorbid physical health problems is similar in SMI and non-SMI patients. Consideration of socio-demographic differences between SMI and non-SMI patients is important in the assessment of excess physical morbidity among SMI patients; in particular, age, and to a lesser extent, ethnicity. Taking into account the cross-sectional nature of the current study - if one considers the literature suggesting that excess mortality is linked to excess physical comorbidity - this study may indicate that having accounted for socio-demographic factors - it is unlikely that cancer, hypertension, coronary heart disease, or atrial fibrillation are responsible for the reduction in life expectancy among SMI patients, though it is possible that under-diagnosis and/or premature death due to these conditions may lead to an underestimation of their prevalence among SMI patients. Any specific effect of certain cancers, in particular lung cancer that may be related to excess smoking among SMI patients could not be assessed since a breakdown of cancer diagnoses was not available. Of the conditions that remained associated with SMI status following adjustment for socio-demographic characteristics, only diabetes was among the most common comorbid condition states identified, suggesting it may have an important role in explaining the mortality gap. The study also suggests that health risk behaviours may be influential mechanisms accounting for excess morbidity, and further research addressing a wider range of determinants (alcohol/drug use, exercise, diet etc.) would help to determine the extent to which each contributes to excess physical morbidity.

## Competing interests

The authors declare that they have no competing interests.

## Authors’ contributions

CW participated in the design, analysis and drafting of the manuscript. PS and MA participated in the data collection and drafting of the manuscript. MH participated in the design and drafting of the manuscript. All authors approved the final manuscript.

## Pre-publication history

The pre-publication history for this paper can be accessed here:

http://www.biomedcentral.com/1471-2296/15/117/prepub
